# Serine Mutation on Amicyanin Reveals Functional and Structural Roles of Asn47 and Asn54 in the Cu-Binding Ligand Containing Loop

**DOI:** 10.4014/jmb.2509.09046

**Published:** 2025-12-15

**Authors:** Eunjeong Kim, Hyojin Jeong, Heejin Nam, Seounghoon Jeoung, Jaeyeong Shin, Moonsung Choi, Sooim Shin

**Affiliations:** 1 Interdisciplinary Program of Bioenergy and Biomaterials Graduate School, College of Engineering, Chonnam National University, Gwangju 61186, Republic of Korea; 2 Department of Biotechnology and Bioengineering, College of Engineering, Chonnam National University, Gwangju 61186, Republic of Korea; 3 Department of Optometry, College of Energy and Biotechnology, Seoul National University of Science and Technology, Seoul 01811, Republic of Korea; 4 Department of Biomaterial Convergence, College of Engineering, Chonnam National University, Gwangju 61186, Republic of Korea

**Keywords:** Amicyanin, Cu center, cupredoxin, electron transfer, redox potential, protein engineering

## Abstract

Amicyanin is a type 1 Cu protein that mediates electron transfer between methylamine dehydrogenase and cytochrome c-551i in *Paracoccus denitrificans*. In this study, a Ser mutation was introduced at either Asn47 or Asn54 located in the Cu-binding ligand loop containing His53 to determine their role in amicyanin functionality. Their spectral and redox properties, and protein stability according to temperature variance and oxidative stress were investigated. N47S amicyanin indicated similar redox potential and stability to native amicyanin. The reaction kinetic of N47S amicyanin toward methylamine dehydrogenase exhibited a similar electron transfer rate, but immensely improved binding affinity compared to native amicyanin. For N54S amicyanin, it attained a more positive redox potential and greatly reduced stability. N54S amicyanin also altered the reaction kinetics with increased electron transfer and decreased binding affinity. Combined with these results, computational simulations of the N47S and N54S mutations suggest that the Ser substitution at Asn54 alters the geometry of the Cu active site by changing the surrounding H-bond pattern. On the other hand, although N47S did not affect the active site, it can be deduced that the position of Asn47 in the loop is significantly altered to influence the interaction of amicyanin with MADH. Hence, we conclude that Asn47 takes charge of the amicyanin affinity for MADH while Asn54 regulates the electron transfer by altering the redox midpoint potential of the active site.

## Introduction

Metal ions and organic compounds are often present in proteins as cofactors for participating in biological reactions, including respiration, electron transfer (ET), and catalysis [1-3]. Proteins containing metal ions are referred to as metalloproteins, among which amicyanin contains a single Cu ion in its catalytic center [4, 5]. Cu-containing proteins are classified as type 1, 2, or 3 depending on the number of Cu ions and the geometry of the ligands interacting with them [6]. Cupredoxins such as azurin, rusticyanin, and amicyanin, which are relatively small proteins with a Cu^+^ ion at the center spanning a broad range of redox midpoint potential (*E*_m_) values, act as an ET shuttle between proteins [7-9]. Especially, the Cu center is coordinated by two N atoms from two His residues and an S atom from one Cys residue in an approximately trigonal planar arrangement with a weak axial ligand usually provided by an S atom from one Met residue [10, 11].

Amicyanin is a cupredoxin present in *Paracoccus denitrificans* that is responsible for mediating the ET from methylamine dehydrogenase (MADH) to cytochrome c-551i [12]. It comprises a 12 kDa-sized β-barrel cupredoxin with two β-sheets consisting of nine β-strands [13]. The Cu center of amicyanin is coordinated by two His (His53 and His95), one Cys (Cys92), and one Met (Met98) residue. The charge transfer transition S(Cys)p → Cu(II)_dx2-y2_ gives rise to the band at 595 nm exhibiting intense blue color of oxidized form of amicyanin [14-16].

**Fig. 1:**
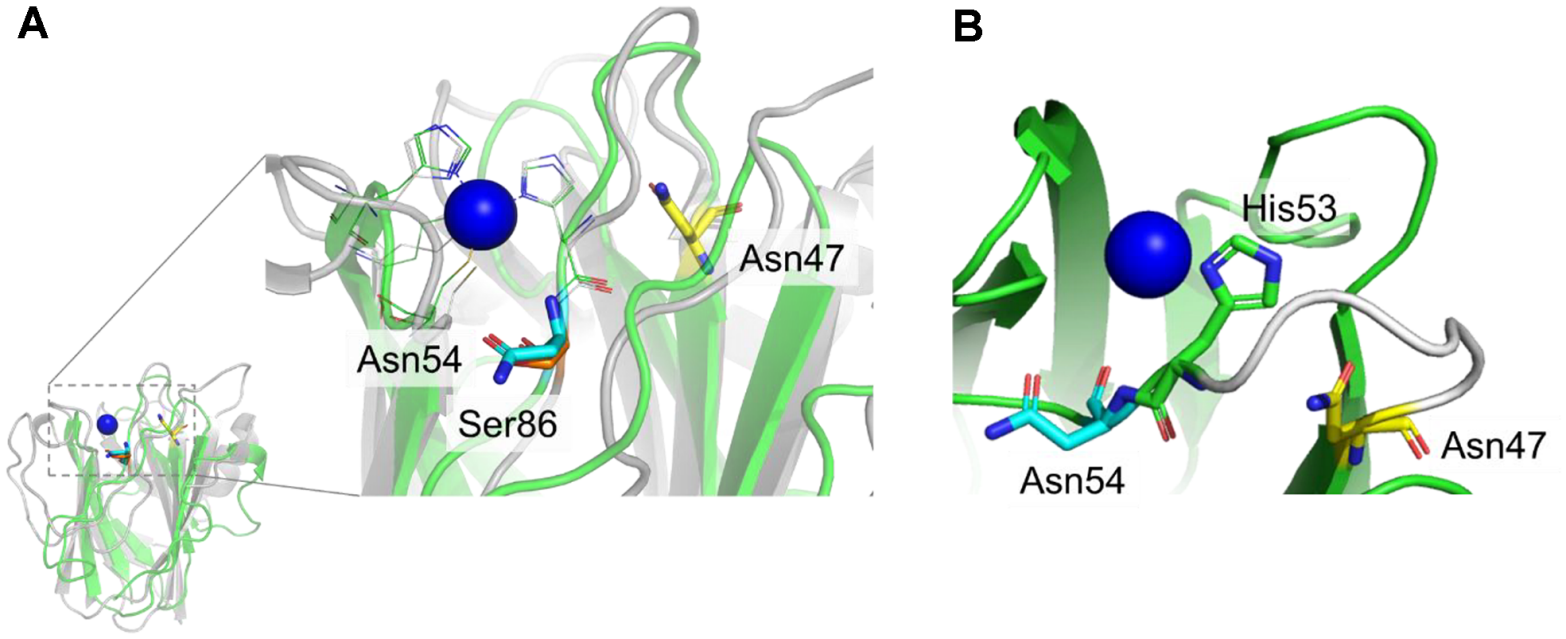
Schematic representation of the structures of amicyanin (green, 1AAC) and rusticyanin (gray, 1RCY). (**A**) is overlapping structure of the overall structure and represents a zoomed-in representation of the gray-dashed box. (**B**) is representation of location of Asn47, Asn54, and His53 in the same loop of amicyanin. Asn54 (cyan stick) in amicyanin is at the same location as Ser86 (orange stick) in rusticyanin. Asn47 is located at the same loop as Asn54 and His53 (green). The loop is colored white. The active sites of amicyanin and rusticyanin are represented by green and gray lines, respectively.

Protein crystallography has revealed that the residues near the ligands interacting with the Cu center can alter the redox potential and the stability of the Cu center [17-19]. Especially, various mutations at the Ser86 residue around the Cu center in rusticyanin increase the *E*_m_ value and Cu stability under acidic conditions compared to the native protein [20], which indicates that the Ser residue around Cu site could play an important role in the change in *E*_m_. This piqued our interest in the influence of the Ser residue in the Cu-binding ligand loop in other cupredoxins. When comparing the structures of rusticyanin and amicyanin, Asn54 in the latter is in the same location as Ser86 in the former (Fig. 1A, Table 1). Furthermore, it is expected that Asn54 plays a role in tuning *E*_m_ value by influencing adjacent His53 which is one of the Cu-binding ligands (Fig. 1B). In addition to this, we also focus Asn47, the other Asparagine located in the same loop as Asn54. Serine is similar in size and hydrogen-bonding ability to asparagine but slightly less polar, and is therefore expected to be suitable for examining subtle effects on electron transfer without disrupting structural stability. In the present study, a Ser mutation was introduced at Asn54 or Asn47 in the Cu-binding ligand loop of amicyanin via rational site-directed mutagenesis. Subsequently, the effects of the Ser mutations on the thermal stability of the Cu center and the electrochemical properties of amicyanin were investigated.

## Materials and Methods

### The Site-Directed Mutagenesis of Amicyanin

Either the Asn47 or Asn54 residue in amicyanin was substituted with Ser via site-directed mutagenesis using the *mauC* gene with customized primers (Macrogen, Republic of Korea). The amicyanin gene was cloned between HindIII and EcoRI in pUC19 vector for both site-directed mutagenesis and protein expression. To construct N47S mutated amicyanin, the forward primer sequence was 5'- ACC TGG ATC TCA CGC GAG G -3' and the reverse primer sequence was 5'- GAC GGT GTC GCC GAC CT -3'. To construct the N54S variant, the forward primer sequence was 5'- ATG CCG CAC TCA GTC CAT T -3' and the reverse primer sequence was 5'- CGC CTC GCG GRR GAT CT –3'. Each primer was dissolved in Tris-EDTA (TE) buffer to provide a concentration of 200 pmol/μl. Site-directed mutagenesis was performed via the polymerase chain reaction (PCR) using a T100 Thermal Cycler (Bio-Rad Laboratories, Inc., USA) at annealing temperatures of 60.5°C for N47S and 58.2°C for N54S. To remove the methylated template, the product was then incubated with DpnI (Invitrogen, USA) overnight at 37°C.

### Preparation of the N47S and N54S Amicyanin Variants

After amplification using DH5α, the plasmid was transformed into BL21(DE3) for protein overexpression. The overexpression of each variant was accomplished by adding isopropyl β-D-1-thiogalactopyranoside (IPTG) to 1 L of Luria-Bertani (LB) medium containing 100 μg/ml of ampicillin. Pre-culturing was performed in 4 ml of LB medium with 100 μg/ml of ampicillin at 30°C for 4 h. Afterward, 200 μl of the incubate was inoculated in 1 L LB medium containing 0.03 g of copper sulfate pentahydrate (CuSO_4_∙5H_2_O) with the same concentration of antibiotics. After culturing overnight at 37°C, protein overexpression was performed at 30°C for 4 h by adding isopropyl β-D-1-thiogalactopyranoside (IPTG) to a final concentration of 0.5 mM. After induction, the cells were harvested by centrifugation at 14,391 g for 30 min at 4°C, and the wet cell weight was then measured. Based on the wet cell weight, the cell pellet was resuspended in SET buffer (500 mM sucrose, 0.85 mM ethylenediaminetetraacetic acid (EDTA), and 200 mM Tris-HCl at pH 7.5). Two drops of 1 M magnesium chloride (MgCl_2_), 20 μl of DNase, 0.04 g of lysozyme, and 60 μl of 100 mM phenylmethylsulfonyl fluoride (PMSF) were added to the resuspended cell solution. After incubation for 10 min at 30°C, the cells were two-fold diluted with distilled water and incubated for 1h at 30°C. The periplasmic fraction and residual fractions were separated by centrifugation at 14,391 g for 30 min at 4°C. For titration of CuSO_4_, the periplasmic fraction was incubated in a water bath at 30°C for 10–15 min. Afterward, 1 M CuSO_4_ was added to the periplasmic fraction until the final concentration of the former reached 600 μM.

Protein purification was accomplished via fast protein liquid chromatography (FPLC) (Bio-rad Laboratories Inc., USA) using a Ni-nitrilotriacetic acid (NTA) column. The CuSO_4_-treated periplasmic fraction was centrifuged at 14,391 g for 40 min at 4°C to eliminate aggregation in the solution. The clear blue-colored solution was loaded onto the Ni-NTA column and amicyanin was solely purified with various buffers. Beforehand, 50 mM potassium phosphate (KPi) buffer containing 10 mM imidazole and 300 mM NaCl at pH 8.0 was passed through the column to eliminate the non-specifically bound proteins. Afterward, amicyanin was eluted with 50 mM KPi buffer containing 100 mM imidazole with 300 mM NaCl at pH 8.0. The eluted amicyanin was concentrated by using 10 K-cut Amicon ultra centrifugal filters and the buffer was exchanged with 10 mM KPi buffer at pH 7.5 to remove the imidazole and salt. After purification via immobilized-metal affinity chromatography, the sample was purified on a diethylaminoethyl (DEAE) column with 10 mM KPi buffer at pH 8.0. The eluted fractions were concentrated by using 10K-cut Amicon ultra centrifugal filters and the buffer was changed with 50 mM Tris-HCl at pH 7.5. UV/Vis spectroscopy (UV-2600, SHIMADZU, Japan) was performed to determine the purity of the variants. Typically, an A_280_/A_595_ ratio of 4 to 1 indicates pure amicyanin. Furthermore, the protein amount was calculated by using the Lambert-Beer equation with the known extinction coefficient of oxidized amicyanin (4.6 mM^-1^cm^-1^). After adding 5% glycerol, the protein solution was stored at -80°C.

### Determining the E_m_ Values of the Amicyanin Variants

The redox properties of amicyanin and the variants were determined via titration against ascorbate and ferricyanide in a glass microelectrode (MI-800/710, Microelectrodes Inc., USA). Beforehand, the microelectrode was calibrated using quinhydrone solution (a 1:1 mixture of hydroquinone and benzoquinone) as the standard, which provided an *E*_m_ value of +286 mV at pH 7.0. After calibration, 30 μM of oxidized amicyanin was titrated against 10 mM of ascorbate in 2 ml of 0.05 M KPi buffer at pH 7.5 and then re-oxidized using 10 mM ferricyanide in the same buffer. The absorbance at 595 nm and the measured redox potential were obtained, after which the midpoint reduction potential (*E^0^*) value was calculated by using the Nernst equation as follows:

E=E0−RT/zFlnaRed/aOx, (1)

where *E^0^* indicates the midpoint reduction potential under standard-state conditions, *R* is the ideal gas constant (8.314 mol^-1^K^-1^), T is the temperature (K), *z* is the number of electrons transferred during the reaction, *F* is Faraday’s constant (95,484.56 C mol^-1^), *a_Red_* is the chemical activity of the relevant species in the reduced form, and *a_Ox_* is its chemical activity in the oxidized form.

### CV Measurements

The redox properties of the amicyanin variants were determined by using a CV (PGSTAT128N, Metrohm Autolab, Netherlands) equipped with an Au working electrode, a Pt counter electrode, and an Ag/KCl reference electrode. Before the measurements, the Au working electrode was polished using alumina powder and sonicated at an amplitude of 25% for a total of 3 min (alternately 20 s on and 10 s off). Finally, the modified Au electrode was obtained by incubating it in saturated aldrithiol-4 solution and then thoroughly rinsing it in distilled water after 30 min.

The redox potential was measured using 26 μM amicyanin in 10 mM KPi buffer at pH 7.5 with a scan rate of 20 mV/s. The potential values were then converted into values against the standard hydrogen electrode (SHE).

### The Steady-State Kinetics of MADH toward the Amicyanin Variants

The amicyanin variants were reacted with MADH to measure the ET rate from MADH to amicyanin. The reaction was monitored by using a UV spectrometer (S-3100, SCINCO, USA) with a temperature controller. In a 1 ml disposable cuvette, 0.018 μM of MADH was mixed with various concentrations of amicyanin (2, 4, 8, 12, 25, 37.5, or 50 μM) and incubated in 0.1 M KPi buffer at pH 7.5 for 5 min at 30°C. After adding 100 μM of methylamine, the absorbance change was monitored for 1 min at 595 nm. To determine the ET rate of amicyanin toward MADH with various concentrations of the former, the initial velocity was calculated in the steady state. The steady-state kinetic parameters were obtained from the results of the reaction from MADH to amicyanin and applied to the Michaelis-Menten equation as follows:

$$V_{0}=V_{\max}\left[S\right]/\left(K_{M}+\left[S\right]\right),$$ (2)

$$k_{cat}=V_{\max}/\left[E_{t}\right],$$ (3)

where *V_max_* indicates the maximum reaction rate at which the substrate becomes saturated, *K_M_* is the Michaelis constant (the substrate concentration at which the reaction rate is half of *V_max_*), [S] is the substrate concentration, and *k_cat_* is the turnover number (the number of substrate molecules turned over per unit time) by each enzyme molecule.

### Thermal Stability Measurements of Native Amicyanin and the Variants

To demonstrate the thermal stability of the amicyanin variants, 18 μM of amicyanin was incubated in 0.1 M KPi buffer at pH 7.5 while varying the temperature from 20–70°C at 1°C/min. The thermal stability depending on the temperature was measured using a UV spectrophotometer (UV-2600, SHIMADZU, Japan) with a CPS-100 temperature controller.

### Measurement of the Intramolecular ET in the N47S and N54S Amicyanin Variants

To estimate the ET from Cu^2+^ in the active site of amicyanin to Co^2+^ in the His-tag, Co was first loaded onto the His-tag of the amicyanin variants. 30 μM of N47S or N54S amicyanin was incubated with 100 μM of cobalt chloride (CoCl_2_) in 50 mM Tris-HCl buffer at pH 7.5. The absorbance at 330 nm was measured for 30 min with intervals of 1 min using a UV/Vis spectrophotometer (UV-2800, SHIMADZU). Unbound Co was completely eliminated via buffer exchange using 50 mM Tris-HCl at pH 7.5.

To demonstrate the intramolecular ET in amicyanin, 30 μM of the Co-loaded amicyanin variant was reduced by using a stoichiometric amount of ascorbic acid (Sigma-Aldrich, USA) in 50 mM Tris-HCl at pH 7.5. After monitoring the pattern of the maximum absorbance centered at 595 nm, 5 mM H_2_O_2_ was added to oxidize Co^2+^ on the His-tag of the mutated amicyanin. The oxidation of Cu^1+^ to Cu^2+^ was monitored by measuring the absorbance at 595 nm for 25 min with intervals of 30 s.

### Computational Simulation

The Cu-binding ligand loop mutagenesis of the N47S and N54S mutations was simulated by using BioLuminate software (Schrödinger, LLC, NUSA). Subsequently, the H-bonds in the native and mutated amicyanin structures were predicted and the amicyanin structures were aligned.

## Results

### The Spectroscopic Properties of the N47S and N54S Amicyanin Mutants

**Fig. 2:**
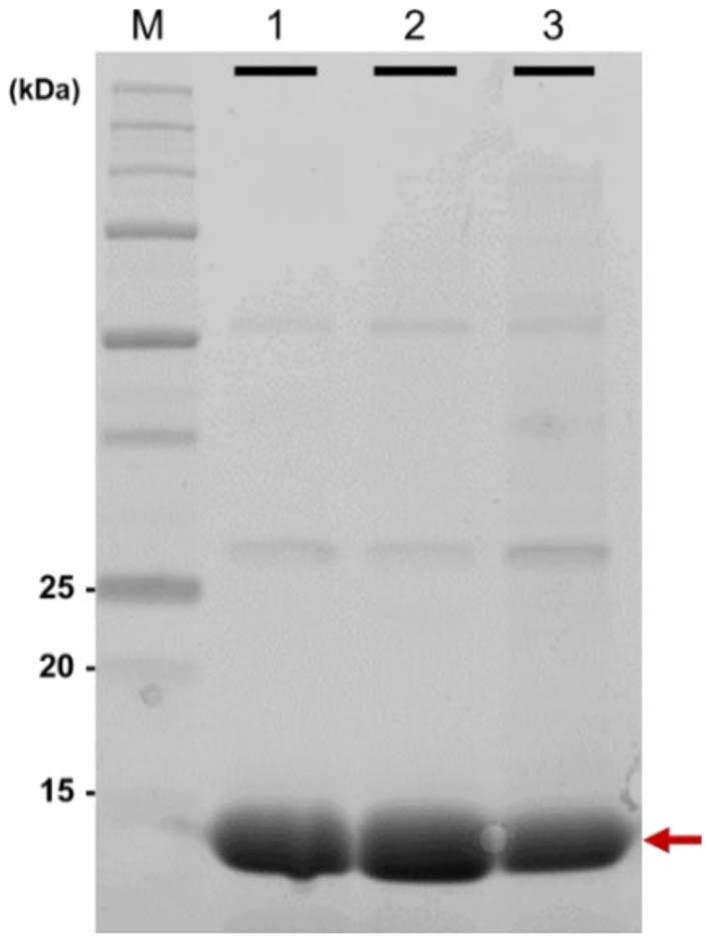
A photographic image of an SDS-PAGE gel showing native, N47S, and N54S amicyanin (lanes 1, 2, and 3, respectively).

**Fig. 3:**
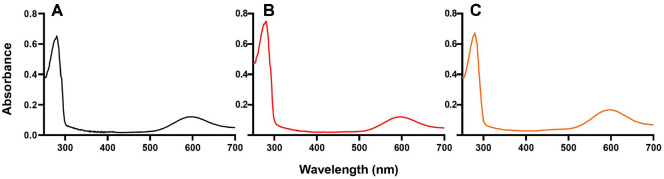
UV/Vis absorbance spectra of (A) native amicyanin, (B) N47S, and (C) N54S.

Single intense bands in a sodium dodecyl sulfate (SDS)-polyacrylamide gel electrophoresis (PAGE) gel indicate the presence of the pure N47S and N54S amicyanin variants. Moreover, their visible absorption spectra were the same as native amicyanin (Fig. 2). Maximum absorbance was centered at 595 nm with a 280 nm/595 nm (A_280_/A_595_) ratio of 4 to 1 (Fig. 3 and Table 1). The protein yields of amicyanin and the variants were calculated by using the Lambert-Beer equation with an extinction coefficient of 4.6 mM^-1^cm^-1^ at 595 nm wavelength and a molecular weight of 12.5 kDa (Table 2). The yields of the N47S and N54S variants were 6.6 and 7.8 mg/g-cell, respectively, which were less than that of native amicyanin (9.0 mg/g-cell). These results indicate that mutations at the N47 and N54 residues affect the yield of amicyanin without changing the geometric properties of its catalytic site.

### The Electrochemical Properties of the N47S and N54S Amicyanin Mutants

**Fig. 4:**
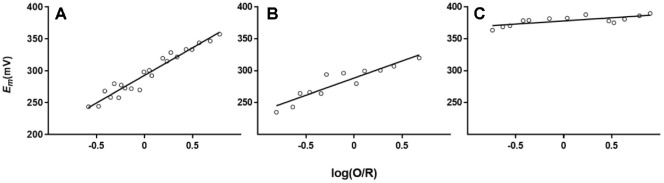
Nernst plots of the spectrochemical titration of native (A) N47S (B) and N54S (C) amicyanin. They were titrated against 10 mM ascorbate (reductant) and ferricyanide (oxidant). The log(O/R) values were obtained from the absorbance change at 595 nm.

**Fig. 5:**
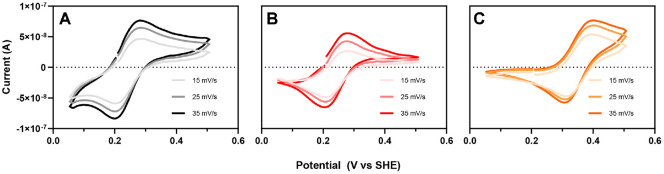
Cyclic voltammograms of native (A) N47S (B) and N54S (C) amicyanins.

The redox properties of the amicyanin from N47S and N54S were determined via spectrochemical titration (Figs. 4 and 5). Amicyanins were titrated with ascorbate (reductant) and ferricynide (oxidant) which both proceed one-electron transfer mechanism [21]. The equivalent of amicyanins were calculated based on the volume of the reductant and oxidant taken. During the reduction, while the equivalent of native amicyanin was 1.33 eq/mol and that of N47S amicyanin was 1.25 eq/mol, N54S amicyanin had low equivalent, 0.57 eq/mol. Likewise, the oxidation of amicyanins showed similar tendency, native and N47S amicyanins exhibited similar values, 1.33 and 1.25 eq/mol. However, N54S significantly decreased the equivalent to 0.63 eq/mol. The redox potential (*E*_m_) value of the amicyanin variants were also obtained during the spectrochemical titration by fitting to the Nernst equation (Fig. 4 and Table 3). The *E*_m_ values of N47S and N54S exhibited a 6 mV decrease and a 90 mV increase, respectively, compared to native amicyanin.

In addition, the *E*_m_ value of the amicyanin variants were determined via cyclic voltammetry (CV). However, the *E*_m_ values obtained by CV method were slightly lower than those determined by spectrochemical titration, a phenomenon commonly observed due to quasi-reversible electron transfer and protein-electrode coupling in protein-film voltammetry, which can bias the apparent midpoint relative to equilibrium titrations [22]. The cathodic and anodic peaks of both variants were separated by approximately 60 mV while the increase in current dependent on the scan rate indicates a reversible response at the aldrithiol-4-modified Au electrode (Fig. 5). The *E*_m_ value of native amicyanin was 236.7 mV, which is close to the reported value (234 mV) [23, 24]. Meanwhile, the *E*_m_ value of N54S was +111.1 mV more positive while that of N47S was slightly more positive compared to that of native amicyanin. This is consistent with the results from the spectrochemical titration experiment (Fig. 4 and Table 2). These results demonstrate that the N47S mutation does not impact the redox potential of the Cu ion in the active site. Furthermore, the increased *E*_m_ value of N54S amicyanin indicates that Asn54 could facilitate electron acceptance from MADH.

### Changes in the Steady-State Kinetics of MADH with Amicyanin due to the Ser Mutations

**Fig. 6:**
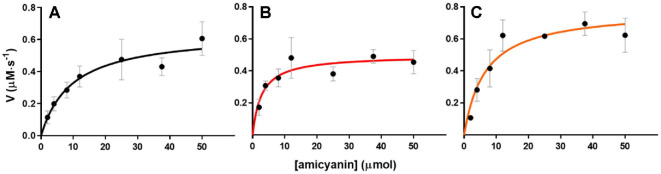
Steady-state kinetics MADH toward (A) Native, (B) N47S, and (C) N54S amicyanins. The data were fitted by using Eq. (2). The points indicate the mean ± standard error of the mean. All of the experiments were performed in triplicate.

As the amicyanin-MADH reaction is known to obey Michaelis-Menten kinetics, the steady-state kinetics of MADH toward amicyanin and its variants were examined via the methylamine-dependent reduction of amicyanin (Fig. 6) [25]. The turnover number (*k_cat_*) values for N54S and N47S were 47 s^-1^ and 28 s^-1^, respectively, which are respectively higher and slightly lower than that of amicyanin (35 s^-1^) (Table 4). This is consistent with the redox potential result for N54S, inferring that it can more easily and quickly accept electrons from MADH than native amicyanin. Furthermore, N54S attained a similar *K_m_* value to native amicyanin whereas that of N47S was significantly decreased (Table 4). This indicates that Asn47 is involved in the binding of amicyanin to MADH.

### Intramolecular ET from Cu^2+^ in the Active Site to Co^2+^ on the 6xHis-Tag of Amicyanins

**Fig. 7:**
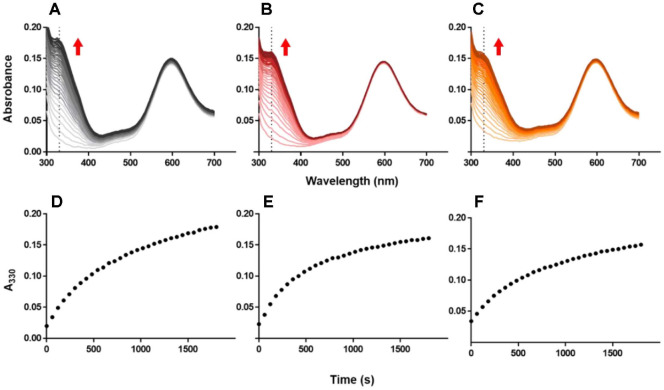
Changes in absorbance due to Co-binding to the 6xHis-tag of (A) native amicyanin, (B) N47S, and (C) N54S amicyanins and after subtracting the initial absorbance from the final absorbance at 330 nm for (D) Native amicyanin, (E) N47S, and (F) N54S.

Intramolecular ET between the Cu active site and the newly engineered redox center was investigated to explore the influence of the mutations on the ET capacity of Cu active site. 6xHis tag is widely used for protein purification with the affinity for divalent metal ions such as Ni^2+^ and Co^2+^. The other application of 6xHis tag as an active site for ET is described in previous research [26, 27]. By the affinity of 6xHis tag with divalent metal, Co^2+^ ions were loaded onto the tag of N47S, N54S and native amicyanins, and the intramolecular ET between the novel and original Cu active site was measured. The generation of Co-redox center was confirmed by observing visible absorption spectroscopy at 330 nm [27] (Fig. 7A-7C, respectively). The Co-binding capacity of the 6xHis-tag was measured by using spectrochemical titration and then calculating the spectral change at 330 nm (Fig. 7D-7F). The binding capacities of N47S and N54S were identical to that of native amicyanin, which suggests that the Ser mutation at either Asn residue did not affect the formation of the Co^2+^ loaded 6xHis-tag redox-active site.

**Fig. 8:**
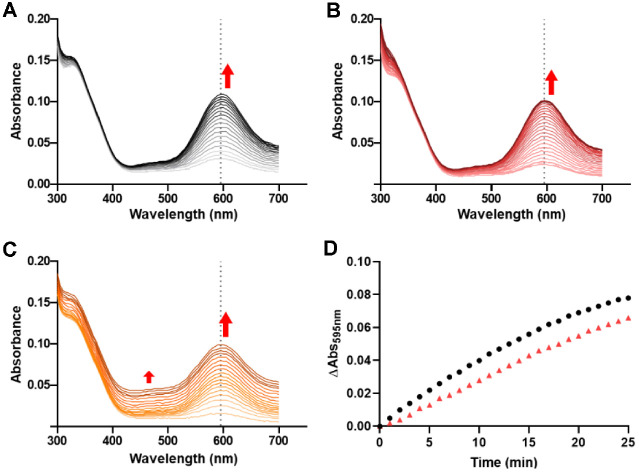
Spectral changes of Co-binding 6xHis-tagged amicyanins as intramolecular ET proceeds. (**A**) native, (**B**) N47S, (**C**) N54S. Absorbance change at 595 nm versus time of native (black), N47S (red) amicyanins are indicated in (**D**).

After the reduction of the Cu ion in the active site, the intramolecular ET was initiated by the oxidation of Co^2+^ to Co^3+^ using hydrogen peroxide (H_2_O_2_). ET was monitored by recording the change in absorbance at 595 nm (Fig. 8A-8C). During the process, Cu^1+^ ion is re-oxidized by the ET to Co^3+^ and the 595 nm peak is recovered. N47S amicyanin decreased the intramolecular ET rate, consistent with the result of a reduced ET rate in steady-state kinetics of MADH (Fig. 8D). For N54S, it was expected that the intramolecular ET rate would increase due to more positive *E*_m_ value, but exact intramolecular ET rate for N54S was not obtained due to baseline shift at overall spectrum (Fig. 8C). This finding reinforces the result of the thermal stability test whereby both indicate that the Ser mutation at the Asn54 residue destabilizes the Cu-binding site in amicyanin.

### The Thermal Stability of the Amicyanin Variants

**Fig. 9:**
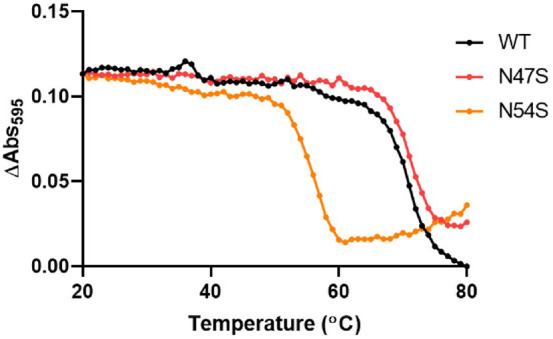
Changes in UV/Vis absorbance according to temperature for thermal stability testing. Absorbance change at 595 nm versus temperature of native (black), N47S (red), and N54S (orange) amicyanins are indicated in the graph.

The thermal stability of the mutated and native amicyanin (as the control) were determined by UV/Vis spectroscopy with the wavelength range from 250–700 nm (Fig. 9). To understand the influence of the N47S and N54S mutations on Cu active site, change of the 595 nm absorbance which arises from strong interaction between Cu^2+^ and Cys ligand in distorted tetrahedral geometry of type 1 Cu site was monitored [14]. The melting temperature (*T*_m_) of native amicyanin was 69.92°C (Fig. 9 and Table 5). N47S was 70.93°C which is 1 degree higher than native, however, N54S significantly decreased in the *T*_m_ to 53.57°C. This is because the Ser mutation at the Asn54 residue, which is located near one of the Cu-binding ligand residues (His53), affects the geometry of the active site in amicyanin.

### Computational Simulation of Structural Change by N47S and N54S Mutations

**Fig. 10:**
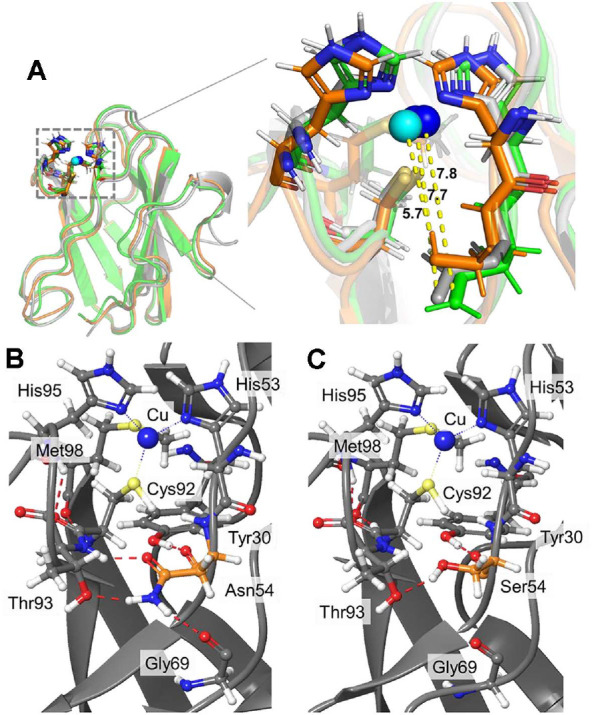
Schematic representation of the alignment of N47S (green) and N54S (orange) compared to native amicyanin (gray, 1AAC) and H-bond prediction. (**A**) is the overall structure and a zoomed-in schematic of the graydashed box. The predicted distances between Cu and position 54 are indicated as yellow-dashed lines. The distance from Cu to Ser54 OG atom is 5.7 Å in N54S. The distances from Cu to Asn54 ND2 atom are 7.7 and 7.8 Å in native and N47S amicyanins, respectively. The stick and ball models indicate the residues composition of the active site while the sphere is the Cu ion in N54S (cyan) and native amicyanin (blue). The Cu ion in the N47S and native amicyanin binding site is in the same position. (**B**) and (**C**) are Predicted H-bonds around active site in native and N54S amicyanins respectively. The H-bonds are denoted as reddashed lines.

The structures of N47S and N54S amicyanins were simulated by BioLuminate software to investigate influence of the Ser mutations on the active site. The structures of native amicyanin and the two predicted variants were compared via alignment using the PyMOL molecular graphics system. From the results, neither variant showed a change in the distance between the ligands and Cu incorporation at the active site. However, although the active site location in N47S was the same as that in native amicyanin, that in the N54S variant had moved away from the original location of native amicyanin. Then, predicted H-bond pattern of mutants were analyzed. H-bond pattern for the N54S variant showed that substituting Asn54 with Ser broke an H-bond between Ser54 and Thr93 near one of the Cu-binding ligand residues, Cys92 (Fig. 10B and 10C). The predicted distance between the Cu and the O-atom of Ser54 side chain in the N54S amicyanin was 5.7 Å, which is shorter compared to the distances 7.7 and 7.8 Å between N-atom of Asn54 side chain in native and N47S amicyanin, respectively (Fig. 10A). It indicates that the active site was moved toward Ser54, thus leading the movement of the Cu with its 4 ligands. In addition, the overall structure of the N54S was loose and the Cu-binding loop was more untangled than the native and N47S ones (Fig. 10A). It supports the findings of lower thermal stability and a more positive *E*_m_ value of N54S amicyanin.

**Fig. 11:**
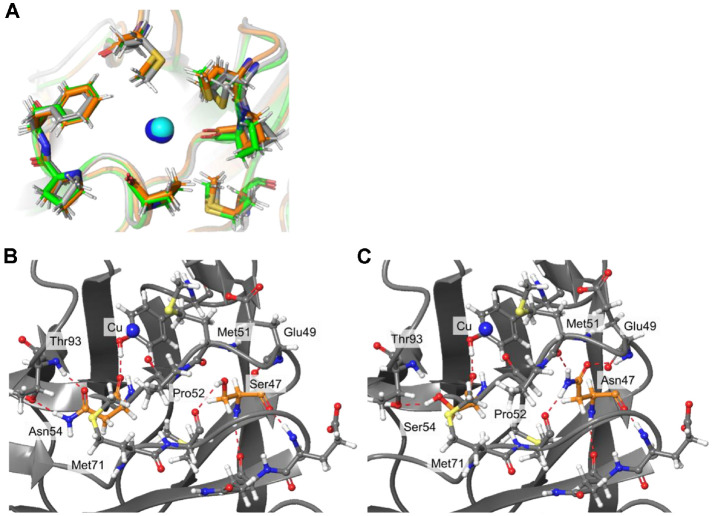
(A) is the alignment of MADH binding region of native (gray), N47S (green), and N54S (orange). The stick models indicate the residues composition of the active site while the sphere is the Cu ion in N54S (cyan) and native amicyanin (blue) (**B**), (**C**) are predicted H-bonds around MADH binding site in N47S and N54S amicyanins respectively. The H-bonds are denoted as red-dashed lines.

Next, the MADH binding region of N47S and N54S variants was analyzed via simulation. The 7 residues with hydrophobic side chains (Met28, Met51, Pro52, Met71, Pro94, Pro96, and Phe97) were included in the MADH binding region surrounding Cu-binding center [28, 29]. The aligned structures of native and variants show that MADH binding region of N47S mutant amicyanin is more altered than that of N54S one (Fig. 11A). The distances between the Cu atom and the residues in MADH binding region of native, N47S, and N54S amicyanins were compared (Table 6). Met51, Pro52, and Met71 of N47S and N54S were highly biased toward the Cu center than native with shorter distance to the Cu). This is because Asn47 interacts with Met51 by H-bond, but Ser mutation breaks the interaction so that Met51 moves to the Cu center (Fig. 11B). In case of N54S, the N54S mutation breaks the H-bond with Thr93. Thus, the Met51 and Pro52 in the same loop as Ser54 can moved toward the Cu (Fig. 11C). This structural alteration in the MADH binding region explains the greater transition of Km of N47S and N54S.

## Discussion

In this study, we demonstrated the impact of a Ser mutation at either Asn47 or Asn54 in amicyanin generated by using site-directed mutagenesis. The functions of each mutated amicyanin variant were investigated by measuring the *E*_m_ value and steady-state kinetics of MADH binding. Moreover, the stability of the variants was demonstrated by measuring their temperature tolerance and intramolecular ET after Co loading onto their His-tags. Although the functionality and stability of the N47S variant were similar to those of native amicyanin, these properties significantly changed in the N54S variant.

The Cu-binding ligand loop of a cupredoxin is significant for interacting with redox partner proteins such as MADH and aromatic amine dehydrogenase (AADH). The sequence, length, and structure of the loop in cupredoxins vary quite considerably, which has been well characterized [30]. Mutagenesis of the Cu-binding ligand loop can alter the functional properties of a cupredoxin. In the case of amicyanin, the P94F and P94A variants provide more positive redox potential values by obstructing the rotation of one of the Cu-binding ligand amino acids (His95) [17]. In addition, the P52G and M51A variants convert the true ET rate into gated ET by altering the interaction with MADH [31]. In the case of rusticyanin, substitution at Ser86 to Asn, Asp, and Gln decreased the redox potential and stability under acidic condition indicating Ser86 is important for great acid stability and high redox potential of rusticyanin. Moreover, this suggests that the redox potential of the active site of blue copper proteins can be modulated by alter the residues not interacting Cu directly [20].

Both Asn47 and Asn54 in amicyanin are located in the loop containing His53, which is part of the ligand that interacts with Cu. Especially, Asn54 shares the same position as Ser86 in rusticyanin in that they are both located adjacent to the Cu-binding ligand [12, 32, 33]. The Ser mutation at the Asn54 residue increased the ET rate and *E*_m_ value. Ser contains a hydroxyl group that can form a short H-bond with another polar amino acid. The P94F and P94A mutations of amicyanin hinder the flipping of His95, so the redox potential becomes more positive [17]. Similar to P94F and P94A, Ser54 could form a short H-bond with His53, thereby altering the redox potential. Furthermore, as a consequence of the increase in *E_m_*, the ET rate from MADH to amicyanin became faster than that of native amicyanin. The N54S amicyanin variant showed highly decreased thermal stability and tolerance toward oxidative stress compared to the native and N47S amicyanin, which could be because Asn54 is adjacent to His53 (part of the Cu-binding ligand in amicyanin) (Fig. 1C). The computational simulations display the influence of N54S to the Cu center. The broken H-bond between Ser54 and Thr93 alter the position of Cys92 which is one of the Cu-binding ligand residues resulting Cu site movement (Fig. 10B and 10C).

Different from N54S amicyanin, the N47S variant had similar spectral, redox, and steady-state kinetic properties but increased *K_m_* compared to native amicyanin. This indicates that only the Ser mutation at Asn47 affected the interaction of amicyanin with MADH. Although the location of Asn47 is further from the Cu-binding ligands than Asn54, it is located at the start of the loop containing the Cu-binding ligands (Fig. 1B), and thus the spectral and redox properties of the N47S variant were the same as native amicyanin. The computational simulations exhibit that N47S drives Met51 and Pro52 to move toward the Cu center (Fig. 11B and 11C) (Table 6). This seems that the absence of the H-bond with Met51 by Ser substitution let the residues to incline toward the Cu center. The former research suggested that Met51 is important surface residue intervening media within the protein complex of MADH and amicyanin [31]. Thus, the role of Asn47 is concluded regulating amicyanin-MADH interaction via modulating position of Met51. This study suggests that the redox potential (or electron transfer) can be modulated via Ser mutation at the residue near the Cu-binding ligands. The N54S mutation increased the *E_m_* and the ET rate while decreasing the thermal stability. The computational simulations further show that the Asn54 influences them via altering the Cu center geometry. Other than Asn54, Asn47 did not affect the *E_m_* and the thermal stability, however, the N47S mutation enhances the affinity with MADH by relocating the residues in the MADH binding region.

This study demonstrates that subtle mutations within the Cu-binding loop of amicyanin distinctly modulate its ET and protein-interaction properties. Asn47 mainly mediates complex formation with MADH, whereas Asn54 modulates redox potential and ET through structural rearrangement around the copper site. These mechanistic distinctions underscore the engineering potential of amicyanin and provides a structural framework for rationally engineering cupredoxins with customized affinities or redox potentials. To this end, these findings are expected to contribute to the development of bioelectrocatalytic systems, direct-electron-transfer (DET) biosensors, and synthetic biological circuits where controlled electron transfer is essential.

## Figures and Tables

**Table 1:** The loop sequences of the cupredoxins.

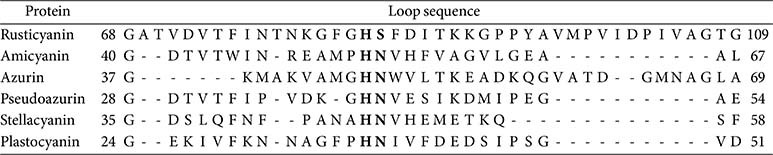

**Table 2:** The A280/A595 absorption ratio and protein yield of native amicyanin and the variants.

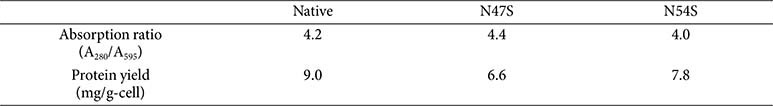

**Table 3:** The redox midpoint potential (*E*_m_) values of native amicyanin and the variants.

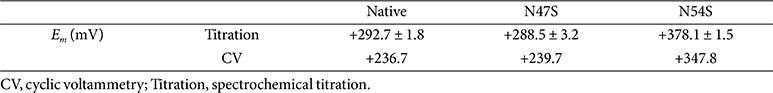

**Table 4:** The kinetic parameters for the methylamine-dependent reduction of native amicyanin and the variants complexed with MADH.

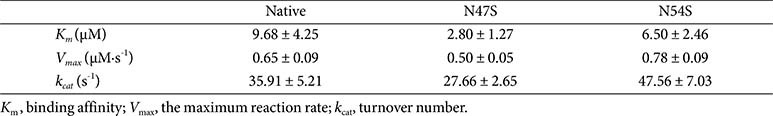

**Table 5:** *T_m_* of native amicyanin and the variants.

	Native	N47S	N54S
*T_m_* (C°)	69.92	70.93	53.57

**Table 6:** The distances of residues in MADH binding region to the Cu center of native, N47S, and N54S amicyanins.

Atom	Native	N47S	N54S
Met28/CE	4.9	5.0	5.0
Met51/CE	8.0	7.0	7.1
Pro52/CG	8.1	6.9	7.1
Met71/CE	5.7	5.0	5.1
Pro94/CG	4.3	4.5	4.5
Pro96/CD	7.4	7.5	7.4
Phe97/Cz	6.4	6.4	6.3
